# A Meteorological Table

**Published:** 1804-12-01

**Authors:** 

**Affiliations:** Brompton


					Meteorological Tabic. 571
A Meteorological Table, by Dr. HiggINs, of Bromptoii.
Days
of the
Month.
i ii-f nu-iRctcr.
Fahrenheit
Height ot tfte Uaro-
meter. Inches.
U c
00
y w}
?2
WEATHER.
zoi59w
21157-jeo
22(56 157
54
52
52
53
5x
,'6
23j4I
24 47
261-2
47
47 |5r
5? 153
5? 60
5? 57
2[49 57
3 46 ;5?
414- 44
40 '41
3 s 4P
,39 :46
^p1 |52
9 4r 44
icUx (56
5?
c6
58
53
44
46
13
?4:5r
J5 43
16 43
!46
45
f ?5?
6.t>6
5-55
3-558
2.22
X.I J
1.11
4 44|
?-55
3 33
?-55
2.77
5-55!
3.88
3-88
0.55
6.66
5 55
4.44
7-77
H.11
6. 6!
13-35j
10 00!
I3-33!
r4-44j
11.66
6.66'
7-77
?-55'
10.00
11.11,
30.04;
29.93
52
72
96
3?
29.97
90
75
40!
30
z9
30,02
29.84!
.48
?76
??9
30.Oi
29-98
?95
?87
?69
?3i
30.04
29.72
?54|
?34
?99
30.00
.00
29* 94j
?77
.58
.67
.96
30.67
.41
.26
?36
9-59
?57
.26
?37
30-2
29.89
75
33.09
33
47
551
?6,
?63
.85
30.30
?7i
?3i
.29
?33
299?|I 7
?52
?56
?39
?94
3|3?.i8
2985
.80
30.19
37;
53i
53
23
.31)14
Fair, with flying clouds.
Ditto, with rain in the night.
Stormy,an Aurora Borealis in theEven.
Fair.
Fair.
Cloudy, rain in the night.
Ditto, rain in the eveniug.
?Showery.
Ditto, with rain in the night.
Rain.
Showery. \
Cloudy, rain in the evening.'
Cloudy, rain at night.
Showery, wind and rain at night.
Windy.
Windy.
Cloudy, with wind.
Cloudy.
Foggy.
Rain.
Foggy, rain at night.
Rain.
Rain.
Showery.
Showery.
Rain.
Small rain.
Small rain.
Cloudy, rain in the night.
Cloudy, with rain at night.
Rain.
Account

				

